# A Rare Case of Concurrent SNRPB Mutation and 22q11.2 Microduplication in a Child With Cerebro‐Costo‐Mandibular Syndrome

**DOI:** 10.1155/crig/4169170

**Published:** 2026-02-19

**Authors:** Elizabeth Slear, Claire Thompson, Virginia Ruas

**Affiliations:** ^1^ Burnett School of Medicine at Texas Christian University, Fort Worth, Texas, USA; ^2^ Department of Pediatrics, Cook Children’s Medical Center, Fort Worth, Texas, USA

## Abstract

We present a unique case of an infant born with both a microduplication of 22q11.2 and SNRPB gene mutations suggestive of cerebro‐costo‐mandibular syndrome (CCMS). Microduplications of 22q11 are known to present with a variety of phenotypes ranging from asymptomatic to significant physical and mental health challenges. CCMS is a rare autosomal dominant condition caused by a mutation in the SNRPB gene and typically presents with posterior rib malformations and branchial arch deformities. There have been less than 100 reported cases of CCMS in the literature, and this may be the first documented case of a patient with both CCMS and a 22q11 microduplication.

## 1. Introduction

Cerebrocostomandibular syndrome (CCMS) is a rare genetic syndrome with less than 100 reported cases in the literature and is characterized by branchial arch malformations and significant rib gaps [1]. CCMS is caused by an autosomal dominant mutation in the small nuclear ribonucleoprotein polypeptide B (SNRPB) gene, which helps produce a spliceosome [2]. The majority of cases of pathogenic variants of the SNRPB gene are sporadic, though familial cases have been reported [2, 3]. Patients typically have a reduced number of ribs, gaps between their ribs, and significant micrognathia [2]. CCMS is associated with Pierre Robin sequence, a sequence in which mandibular hypoplasia leads to abnormal positioning of the tongue and abnormal development of the palate [4].

The prognosis of patients with CCMS is poor, with roughly 20% of patients dying within the first 12 months of life [2]. As per data, looking at cases of CCMS before the year 2000, approximately 50%–70% of infants born with CCMS died within the first year of life, but the life expectancy has increased due to early detection and intervention [2]. Life expectancy is also greatly dependent on the severity of symptoms present, and the more severe cases typically do not live beyond 12 months of age [2]. Children with CCMS often develop additional medical problems during childhood and are most likely to die from respiratory disturbances [1]. Respiratory problems are common in this population, regardless of disease severity, and are often due to upper airway obstruction or reduced thoracic cavity space [2]. CCMS patients often are difficult to intubate and have feeding difficulties along with scoliosis, microcephaly, developmental delays, heart defects, horseshoe kidneys, and cleft palate abnormalities [2].

Duplication of Chromosome 22q11.2 has been known to present with a variety of phenotypes including Pierre Robin sequence [4]. Some patients with 22q11 duplications are completely asymptomatic, while other documented cases have restricted growth, distinct epicanthal folds, vision or hearing deficits, seizure disorders, or deformities of hands or feet [5–7]. It is common for the mothers of these patients to have some type of learning difference or ADHD, but often the parents of these children are asymptomatic [5]. It has been estimated that the prevalence of these mutations is higher in individuals with intellectual disabilities, but that some phenotypes are mild enough that patients may not undergo genetic testing [4].

We present a unique case of an infant born with both a microduplication of 22q11.2 and SNRPB gene mutations suggestive of CCMS. There have been less than 100 reported cases of CCMS in the literature, and this may be the first documented case of a patient with both CCMS and a 22q11 microduplication.

## 2. Case Presentation

We present the case of a female infant who was born in December of 2023 at 38 weeks and 2 days of gestation by cesarean section for severe fetal micrognathia with oropharyngeal obstruction and polyhydramnios as diagnosed on fetal MRI. The fetal imaging was overall consistent with Pierre Robin sequence. The patient’s head and right arm were delivered via vacuum extraction while still connected to placental circulation. At that time, severe micrognathia was noted. The patient was intubated and ventilated. She was then fully delivered, and the umbilical cord was clamped. Apgars were 2 and 7 at 1 and 5 minutes, respectively.

The patient had a normal prenatal ultrasound at 20 weeks of gestation as per the mother’s report. The mother received inadequate prenatal care, and the severe retrognathia was detected 1 week prior to delivery. Genetics was consulted on day of life 3 for a female infant with multiple congenital anomalies including severe retro micrognathia, supraglottic airway obstruction, mild glossoptosis, mandible hypoplasia, and bilateral dysmorphic ribs.

**FIGURE 1:**
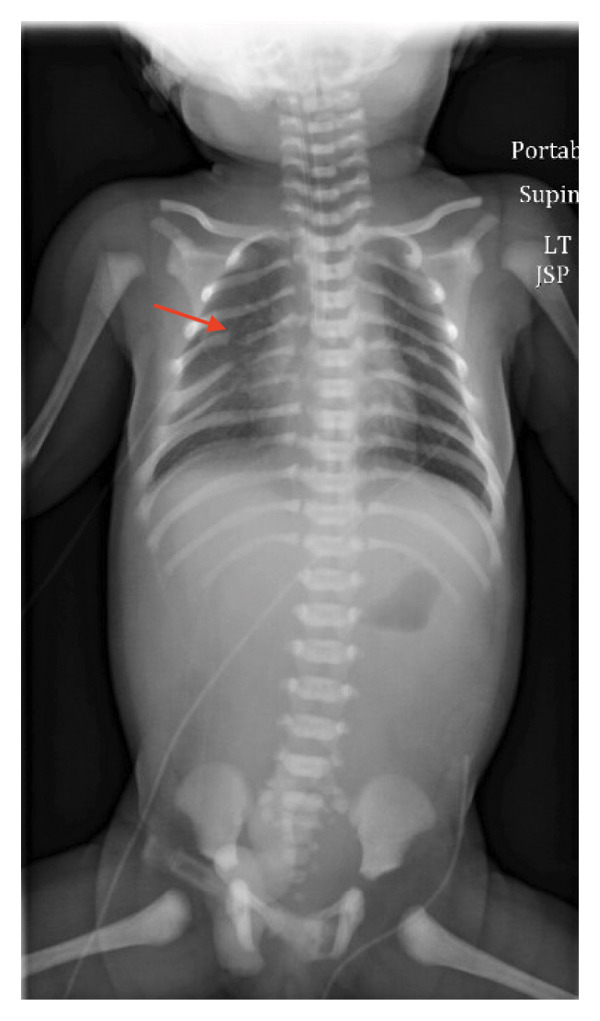
X‐ray demonstrating bilateral dysmorphic ribs with gaps between posterior ribs. A rib dysmorphism is indicated by an arrow.

**FIGURE 2:**
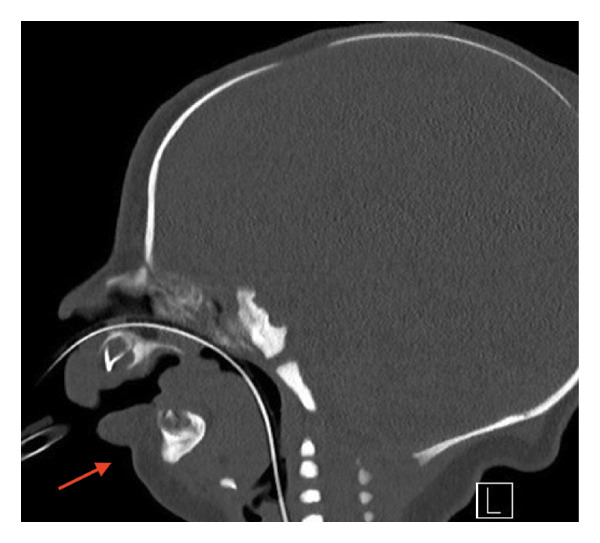
CT scan of facial bones showing significant micrognathia with bilateral hypoplasia of the mandible and mild glossoptosis with narrowing of the posterior oropharyngeal airway and no evidence of bony cleft palate. The micrognathia and mandibular hypoplasia are indicated with an arrow.

Initial imaging revealed bilateral dysmorphic ribs with gaps between posterior ribs (Figure 1). CT of the facial bones revealed significant micrognathia with bilateral hypoplasia of the mandible and mild glossoptosis with narrowing of the posterior oropharyngeal airway (Figure 2). An echocardiogram showed a PDA, which resolved spontaneously by 10 months of age based on a follow‐up echocardiogram. Her initial head and abdominal ultrasounds were normal. On physical exam, the patient was noted to have severe microretrognathia, attached ear lobes bilaterally, long fingers and toes, and facial anomalies consistent with Pierre Robin sequence. The physical exam was otherwise unremarkable.

Early preliminary genetic results were received on day of life 5, and clinical exome sequence analysis showed a heterozygous, pathogenic duplication of 2553 kb within Chromosome 22q11.21. This sequence also indicated a heterozygous variant of unknown significance in the SNRPB gene labeled c.267 + 5G > A. Rapid exome sequencing results on day of life 16 confirmed the patient is heterozygous for a pathogenic duplication of at least 2553 kb within the cytogenic band 22q11.21. This is within the typical duplication region (LCR‐A to LCR‐D). She had a karyotype which revealed a normal female profile of 46, XX.

**FIGURE 3:**
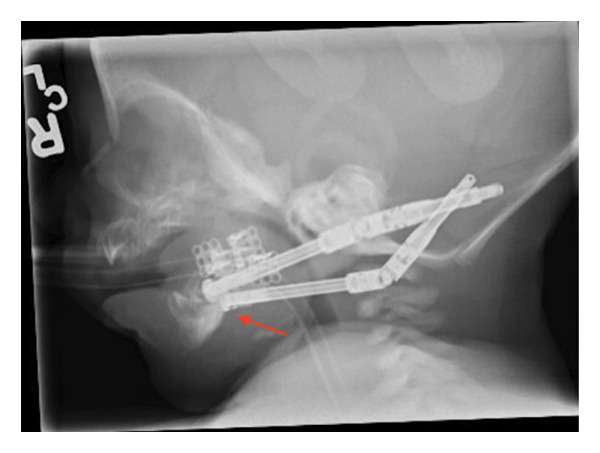
X‐ray of mandible demonstrating postoperative changes following bilateral mandibular distractor placement at 3 weeks of life. The distractors are indicated with an arrow.

At 3 weeks of life, the patient received bilateral mandibular osteotomy distractor placement at Cook Children’s Medical Center (Figure 3). At 4 weeks of life, she underwent replacement of the right mandibular distractor after the posterior footplate of the right‐sided mandibular distractor came off the bone.

She was extubated at 6 weeks of age after completion of distraction with 18.6 mm of total distraction bilaterally.

The patient underwent laparoscopic gastrostomy button with fundoplication at 11 weeks for feeding difficulties, with clinical concern for reflux refractory to medical management, with the need for long‐term feeding access. She was ultimately discharged at 12 weeks of age.

At 7 months of age, she presented to Cook Children’s Medical Center with erythema, swelling, and drainage over the left mandibular hardware site. Bilateral mandibular distractors were removed approximately 1 month prior to the originally scheduled date for hardware removal due to superficial methicillin‐susceptible *Staphylococcus aureus* (MSSA) infection over the left mandibular hardware site.

**FIGURE 4:**
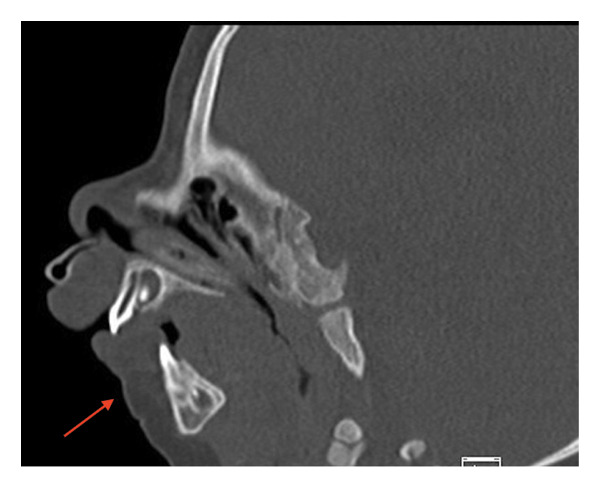
CT scan of facial bones at 10 months of age revealing overall improvement despite recent regression in micrognathia. The improved micrognathia is indicated with an arrow.

She was admitted again at 10 months of age for severe failure to thrive and rhinovirus. She was fed by her gastrostomy tube, and drastic weight improvement was observed while admitted. During the same admission, she was found to have regressed micrognathia. Repeat CT showed nonunion and collapse of previous osteotomies (Figure 4). At the time of her initial surgery, calcifying cartilage was noted. It is unknown why she did not heal after her initial distractor surgery, but it is potentially related to the nature of CCMS, low‐grade infection, malnutrition, or surgical technique. Repeat mandibular osteotomies and mandibular distractors were placed for acute respiratory failure with hypercapnia and regressed micrognathia. She was deemed a good candidate for repeat distraction to address her micrognathia and tongue‐based obstruction contributing to her acute respiratory failure and hypercapnia. She was successfully extubated 1 month after her repeat distraction surgery, and she was distracted to 27.6 mm bilaterally in total. She was discharged on gastrostomy button feeds after a 10‐week admission.

She was seen for a well‐child check at 13 months of age. She was found to be severely malnourished, likely due to an inadequate feeding regimen at home. She had a three‐pound weight loss over the prior 4 weeks, and she was subsequently admitted for failure to thrive. After optimizing her feeding regimen during her 2‐week admission, she was discharged home.

**FIGURE 5:**
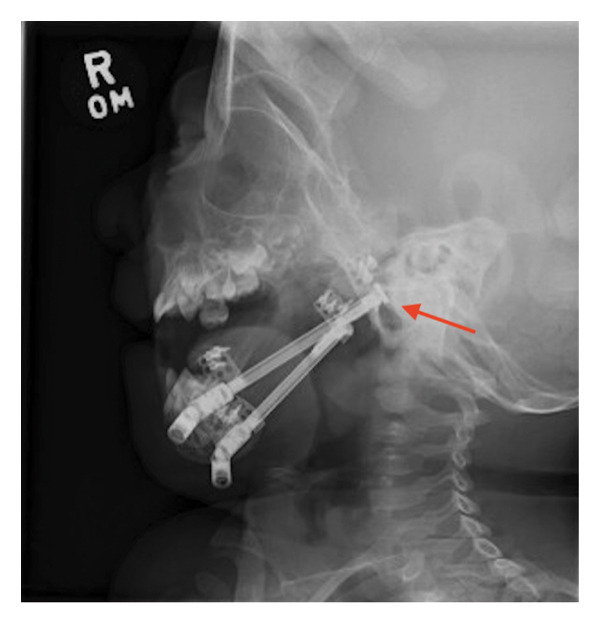
X‐ray of mandible at 15 months of age showing stable postoperative changes following placement of mandibular distraction hardware. Distractor placement is indicated with an arrow.

She was seen at 15 months of age for follow‐up for repeat mandibular osteotomies and distractor placement. As per her mother, she has been eating small amounts of soft food by mouth. Full excursion of distractors is seen in a stable position (Figure 5). Her mandible distractor was removed in September of 2025.

The patient was seen again at the age of 23 months and remained dependent on her G‐tube for feedings. She had been gaining weight appropriately but was noted to have dumping syndrome by the GI department. She had oral aversion. Global developmental delays were noted in addition to abnormal EEG results indicating seizure activity. An appointment with neurology was scheduled, as well as a sedated auditory brainstem response (ABR). A sleep study indicated obstructive sleep apnea.

## 3. Discussion

We present a unique case of a patient with both CCMS due to SNRPB mutation and a duplication of chromosome 22q11. CCMS has been reported less than 100 times in the literature, and this may be the first documented case of a patient with both CCMS and a 22q11 microduplication. After initial intubation at birth, this patient subsequently underwent mandibular distraction osteogenesis for further airway management, which has been shown to be effective for patients with upper airway obstruction due to micrognathia [1, 8]. It is possible that the patient’s survival thus far may be attributable to her unique genetic profile as well as the early interventions she has received to support her airway [2]. By reporting a case with multiple genetic abnormalities and comparing her case to those with singular mutations, it is possible to better differentiate phenotypes that may arise from singular genetic abnormalities. In addition, documentation of the management of this patient, particularly her airway management with intubation during delivery and subsequent mandibular distractor placement, can help guide the management of future patients with CCMS and severe micrognathia. The early and frequent airway interventions she received likely contributed to her prolonged survival. In addition, it is possible that her unique genetic sequence contributes to an improved prognosis, and further cases of similar genetic profiles should be documented in the literature.

## Funding

The researchers did not receive any funding for this research.

## Consent

In accordance with the guidelines of this journal as well as the official policy of Cook Children’s Healthcare System, informed consent was obtained from the patient’s mother to use her anonymized information and images for dissemination in this article. After a comprehensive discussion, she signed a HIPAA authorization form and an image release form.

## Conflicts of Interest

The authors declare no conflicts of interest.

## Data Availability

Data sharing is not applicable to this article as no datasets were generated or analyzed during the current study.
